# Periosteal mitochondria DNA structures drive aging-associated poor skeletal repair

**DOI:** 10.1038/s41413-026-00524-6

**Published:** 2026-04-07

**Authors:** Yanlin Wu, Chuyi Han, Xue Yang, Yitian Wang, Weidong Tian, Quan Yuan, Hui Wang, Haisheng Wang, Bei Yin, Ling Ye, Feifei Li, Fanyuan Yu

**Affiliations:** 1 State Key Laboratory of Oral Diseases & National Clinical Research Center for Oral Diseases, West China Hospital of Stomatology, Sichuan University, Chengdu, China; 2 Department of Endodontics, West China Hospital of Stomatology, Sichuan University, Chengdu, China; 3 Department of Pediatric Dentistry, West China Hospital of Stomatology, Sichuan University, Chengdu, China

**Keywords:** Bone, Bone quality and biomechanics

## Abstract

Insufficient skeletal repair is the primary threat of health span and lifespan in elders with increasingly vast global burden; yet, to date, the knowledge of resolving this crisis remains limited. In this study, we addressed the specific mechanisms underlying aging-associated poor bone repair, which are driven by the mitochondrial DNA structures mitochondrial G-quadruplex (mtG4). We found that mtG4 is spatiotemporal-wisely accumulated within Pdgfra^+^ periosteal mesenchymal stromal/stem cells (PPM) both in healthy and premature aging, which substantially increases cellular senescence and the degenerative alterations of PPM. By utilizing transgenic lineage tracking, PPM organoids formation, mitochondrial transgenic mutation, organoids transplantation, and serial cellular molecular investigations, we reveal that mtG4 in PPM restricts vital mitochondrial genes’ transcription to cause mitochondrial dysfunction, which utterly leads to severe mitophagy and cell senescence. These senescent PPM demonstrates impaired stemness and disrupted fate determination, finally phenocopying aging-associated poor bone repair. This study decodes the mitochondrial genomic reasons for insufficient bone repair during aging, which offers insights for developing cell-type- and disease-specific senolytic therapies in the future.

## Introduction

Insufficient skeletal repair is one of the most threatening challenges of aging,^1–3^ which world-wide causes heavy clinical and socioeconomical burdens each year^1–3^ and has become the top reasons for disability and reduced quality of life in elders.^1–4^ In aged patients, damaged skeletal healing even substantially increases the risks of death.^1–4^ Thus, improving the skeletal repair capabilities during aging is of great importance especially along with the progression of global aging nowadays.^1–4^ Unfortunately, we’re still faced with the huge gap of largely unmet crisis of clinically ameliorated bone repair in aging.^5,6^ To achieve this goal, it is necessary to know the underlying reasons for impaired skeletal repair during aging.^7,8^ Hallmarks of impaired skeletal repair during aging include the disturbed stem cell fate determination, that is the bone-fat imbalance with exacerbated cartilage formation,^7,8^ inflammatory and degenerative mesenchymal stromal cells (MSCs),^9^ accumulation of senescent cells,^10^ and so on. Although more and more reports on these hallmarks emerge, the reasons beneath aging-associated poor skeletal repair remain largely unknown.^10^

Periosteum envelopes the bone surface to fuel cortical bone formation and maintains the integration of bone architecture in homeostasis.^11–13^ Previous studies also evidence the indispensable role of periosteum in guaranteeing bone repair,^13^ and in vivo data have demonstrated the regenerated bone after limb bone fracture being derived from Pdgfra^+^ periosteal MSCs (PPM).^12^ It is known that the histological structure of periosteum changes with aging^14^; however, there still lacking data about the functional alterations of periosteal MSCs in aging and -associated insufficient skeletal repair, neither do we know the underlying mechanisms.^11–13^

G-quadruplexes (G4) are non-canonical secondary DNA structures formed by the self-assembly of guanine-rich sequences via Hoogsteen hydrogen bonds, exhibiting exceptional stability in the presence of monovalent cations, particularly potassium (K^+^).^15,16^ The mitochondrial DNA (mtDNA) is uniquely susceptible to G4 formation due to the high guanine content of its heavy strand, the absence of protective histones, and the prolonged exposure of single-stranded intermediates during replication.^16–18^ Consequently, mtDNA harbors a significantly higher density of potential G4-forming sequences compared to the nuclear genome and is prone to forming unresolved structures within the potassium-rich mitochondrial matrix.^16–18^ These persistent mitochondrial DNA G-quadruplexes (mtG4) act as physical obstacle to polymerases, stalling replication and transcription and inducing genomic instability,^19–21^ a mechanism already related to the aging and degeneration of cutaneous and neuronal tissues.^22,23^ However, whether mtG4 accumulation serves as an upstream driver of periosteum-mediated aging-associated skeletal repair impairment remains to be elucidated.

To this end, in this study we reveal that a unique type of mitochondrial genomic DNA structure, mitochondrial G-quadruplex (mtG4), drives aging-associated poor skeletal repair by causing periosteal MSCs’ mitochondrial dysfunction and cellular senescence. By using the specific detection probe of mtG4, previously established by us,^24^ we herein show the spatiotemporally-specific accumulation of mtG4 in aged periosteum, both in healthy aging and progeria, rather than in other bone compartments. Following this finding, we further demonstrate the substantial elevation of mtG4 in PPM directly causing aging-associated poor skeletal repair in mammals by breaking the fate determination of PPM and meanwhile leading to irreversible cellular senescence. No previous studies have reported the involvement of mtG4 in skeletal aging, neither showed their links in aging-associated poor bone repair. With the utilizations of both in vitro (K^+^ treatment) and in vivo mice model (*Polg* mutation progeria mice model^25–27^: homozygous mutations of DNA polymerase gamma, PolgD257A. Our previous study have established the direct correlation between MSCs senescence with mtG4 accumulation^24^), we prove that increasing mtG4 drives cellular senescence and mitochondrial dysfunctions in PPM, which consequently severely hinders the bone regeneration.

In summary, by using transgenic lineage tracking, transgenic mice, PPM organoids, and comprehensive cellular molecular investigations, we reveal the mitochondrial DNA G4 structures driving cellular senescence of PPM, which severely impairs bone repair in aging. Our data endow us with the insights of underlying mechanisms controlling aging-associated poor skeletal repair, which could furthermore provide with potent targets to enhance bone repair in elders.

## Results

### Periosteum mtG4 arises in both premature and healthy aging

**Fig. 1:**
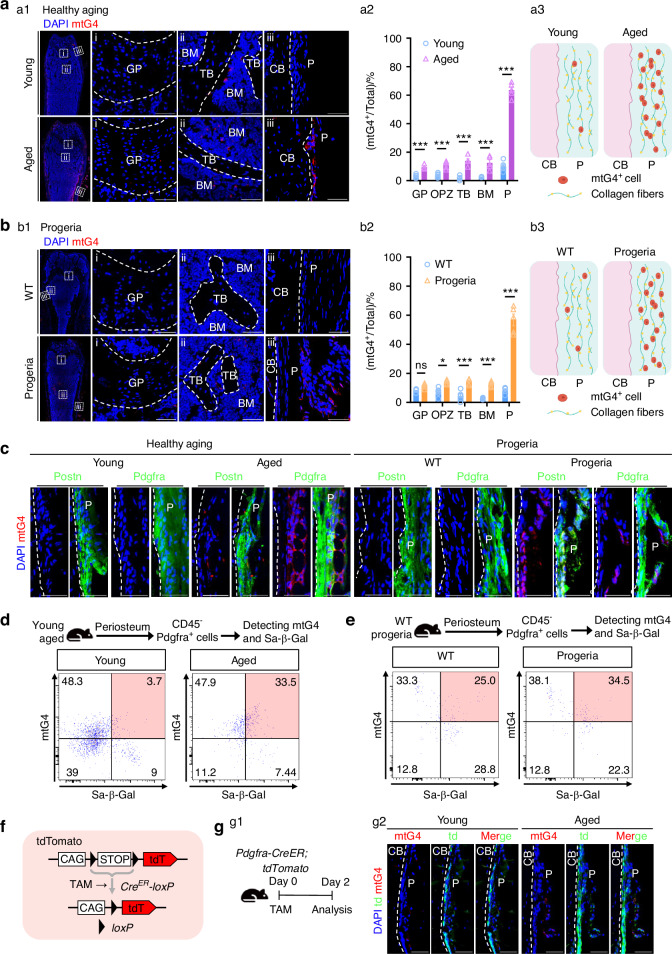
Periosteum mtG4 accumulated in both premature and healthy aging. **a**, **b** Representative IF images showing the alteration pattern of mtG4 during skeletal aging in (a1) healthy aged mice and (b1) premature progeria mice. Quantification of the percentages of mtG4^+^ cells among different skeletal compartments in (a2) healthy aged mice and (b2) premature progeria mice. Schematic of the mtG4 accumulation within periosteum in (a3) healthy aged mice and (b3) premature progeria mice. Scale bar, 50 μm. **c** Representative IF images showing the mtG4 accumulation within the periosteum, delineated by labeling with the periosteum markers POSTN and Pdgfra in healthy aged mice and premature progeria mice. Scale bar, 50 μm. **d**, **e** Representative FCM results showing the dual-labeling with mtG4 and the Sa-β-Gal of Pdgfra^+^ periosteal MSCs (PPM) in (**d**) healthy aged mice and (**e**) premature progeria mice. **f** Schematic of the design and generation of *Pdgfra-CreER*; *tdTomato* mice. **g** (g2) Representative IF images of *Pdgfra-CreER*; *tdTomato* mice showing the accumulation of mtG4 within periosteum and the schematic of this transgenic strategy (g1). Scale bar, 50 μm. GP growth plate, OPZ osteo progenitor zone, TB trabecular bone, BM bone marrow, CB cortical bone, P periosteum, TAM tamoxifen. All experiments were technically replicated three times; *n* = 5 per group. ns no statistical significance. *P < 0.05, **P < 0.01, and ***P < 0.001

By using the reliable mtG4 detection probe-TPA-mTO previously invented by us,^24^ we investigated the alteration pattern of mtG4 during skeletal aging. In our previous research, we have demonstrated that TPA-mTO has high specificity and selectivity for mtG4 structures. It only binds to mtG4, emitting strong red fluorescence, and shows no respond to any other DNA/RNA structures or nuclear genome G4 structures. Moreover, the probe can label mtG4 in various live cell types and fixed tissue cells, making it applicable for immunofluorescence (IF) imaging and flow cytometry (FCM) detection. In this study the “Young” group refers to the young adult mice aged at 2–3 months old (MO), and the “Aged” indicates to the mice aged at 16–20 MO (about 80% were at or above 18 MO), unless stated otherwise. As for the premature aging mice and their wild-type littermates, all mice were aged at 5 MO considering the manifestation progeria in mutant group, unless stated otherwise. Both sexes were used in each group. First, we confirmed the typical skeletal aging hallmark, that is osteoporotic phenotypes, in both trabecular and cortical bone of healthy aging (Fig. S1A, B) and premature progeria (Fig. S1C–E) respectively. IF data next showed that during chronological aging mtG4 levels in femurs were substantially increased (Fig. 1aa1). Among different skeletal compartments, it is obvious that periosteum showing the most up-regulated mtG4 versus all other compartments (Fig. 1aa1). Quantification results furthermore showed that in aged periosteum the proportion of mtG4^+^ cells arising to near 70% (Fig. 1aa2). In the pathological premature aging, we observed the same compartment-specific increasement of mtG4 in periosteum (Fig. 1bb1–2) as that in chronological aging (Fig. 1aa3, 1bb3). Using the periosteum markers, which are Postn and Pdgfra, we confirmed the accumulation of mtG4 occurring within periosteum (Fig. 1c). FCM results quantitatively demonstrated that in both chronologically and prematurely aged mice the mtG4^+^ cells were significantly increased in CD45^-^ PPM (Fig. 1d, e). In combination with the detection of senescence-associated β-Gal (Sa-β-Gal) we demonstrated the significantly increased mtG4^+^Sa-β-Gal^+^ proportion in aged PPM (Fig. 1d, e). Finally, we established the Pdgfra reporter mice, namely *Pdgfra-CreER; tdTomato* mice (Fig. 1f, g), which has been proved to be the golden standard tool of PPM in vivo.^12^ The pulse of *Pdgfra-CreER; tdTomato* mice (Fig. 1gg1) utterly proved the accumulation of mtG4 in aged periosteum, specifically in PPM (Fig. 1gg2).

### mtG4 in PPM positively links with aging-associated poor skeletal repair

**Fig. 2:**
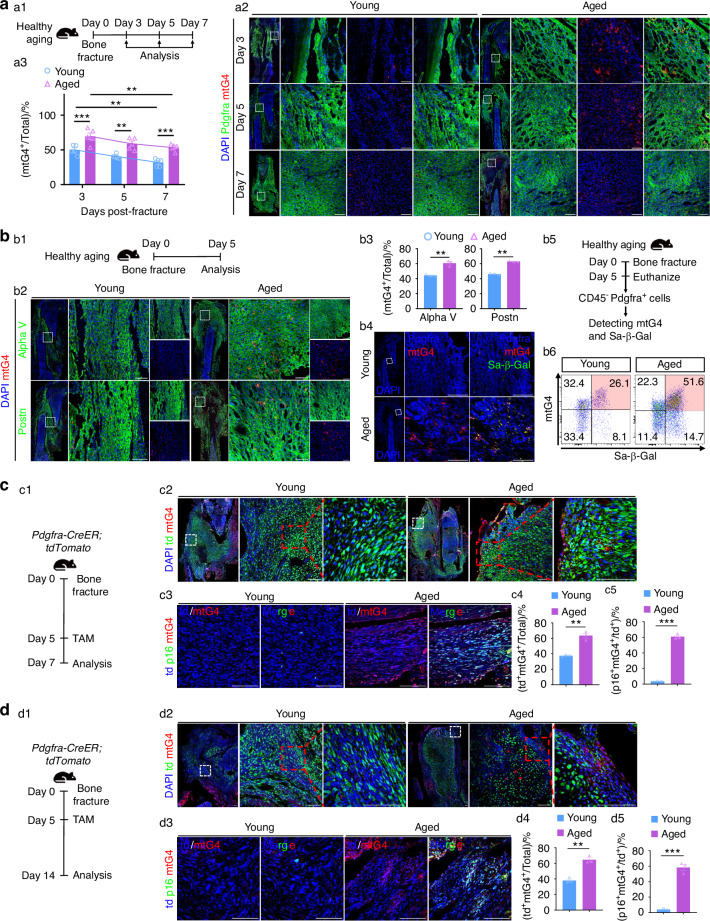
mtG4 in PPM positively links with aging-associated poor skeletal repair. **a** (a1) Schematic of this experiment. (a2) Representative IF images showing the mtG4 accumulation in early callus at d3, d5, d7 post-fracture and the quantification of the percentages of mtG4^+^ cells (a3). **b** (b1) Schematic of this experiment. (b2) Representative IF images of early callus at d5 post-fracture showing the MSCs makers and the mtG4 accumulation. (b3) Statistical data of IF in (b2). (b4) Representative MLIF images of early callus at d5 post-fracture. The image on the left exhibits the dual-labeling with Pdgfra and mtG4, and the right one shows the merged images containing Pdgfra, mtG4, and Sa-β-Gal. (b6) Representative FCM results showing the dual-labeling with mtG4 and Sa-β-Gal of PPM within early callus in healthy aged mice and the schematic of this experiment (b5). **c**, **d** (c1,d1) Schematic of the in vivo tracing of PPM post-fracture. Representative IF images showing the mtG4 accumulation in td^+^ cells at (c2) d7 post-fracture and (d2) d14 post-fracture in healthy aged mice. Quantification of the percentages of mtG4^+^td^+^ cells at (c4) d7 post-fracture and (d4) d14 post-fracture. Representative MLIF images showing the expression of senescence maker *p16*^*Ink4a*^ at (c3) d7 post-fracture and (d3) d14 post-fracture. The image on the left exhibits the mtG4^+^td^+^ cells, and the right one shows the merged images containing td, mtG4, and *p16*^*Ink4a*^. Quantification of the percentages of *p16*^*Ink4a+*^mtG4^+^ PPM at (c5) d7 post-fracture and (d5) d14 post-fracture. Scale bar, 100 μm. All experiments were technically replicated three times; *n* = 3 per group except *n* = 5 in (**a**). ns no statistical significance. *P < 0.05, **P < 0.01, and ***P < 0.001

Our data of micro-CT (µCT) showed that aged murine skeleton owning severely poor repair capabilities (Fig. S2), which was consistent with previous knowledge.^7,8^ In detail, the three-dimensional reconstruction of µCT (3D-RµCT) demonstrated much less regenerated bone in aged mice (Fig. S2Bb1). Quantification of bone volume (BV), bone volume versus total volume (BV/TV), bone mineral density (BMD), trabecular bone thickness (Tb.Th), and trabecular bone number (Tb.N) were all significantly decreased in aged mice, and while the trabecular bone separation (Tb.Sp) was up-regulated (Fig. S2Bb2). Histological data furthermore showed the heavily reduced new bone tissue but increased cartilage tissues in the hard callus of aged mice, but at this timing point, the young mice already owned sufficient new bone formation with reduced cartilage (Fig. S2C). As recent in vivo research has evidenced the indispensable role of PPM in guaranteeing bone repair,^11–13^ we next investigated whether the alteration of mtG4 in PPM also has relation with aging-associated poor repair. At the MSCs infiltration stage our IF data demonstrated that from day 3 (d3) to d5 post-fracture the aged mice harbored significantly more mtG4 in PPM (Fig. 2a). Specifically, at d5 post-fracture those Postn^+^αV^+^ (Itgav, abbreviated as αV) PPM in aged bone showed much increased mtG4 than that in young bone (Fig. 2bb1–3). Multiple labelled IF (MLIF) further revealed that most of the mtG4^high^ PPM in aged periosteum was Sa-β-Gal^+^ senescent cells (Fig. 2bb4). To verify this finding, we harvested the CD45^−^ PPM at d5 post-fracture, respectively from young and aged mice and subjected them into FCM measurements (Fig. 2bb5). FCM quantification data proved that there were much more mtG4^+^ PPM in aged periosteum and meanwhile over 51.6% mtG4^high^ PPM in aged periosteum was Sa-β-Gal^+^ senescent, which was near twice of that in young mice (Fig. 2bb6). To in vivo track the fate of PPM we next utilized the *Pdgfra-CreER; tdTomato* mice. Pulse experiment at the MSCs infiltration stage showed significantly increased mtG4^+^td^+^ PPM in aged mice (Fig. 2cc2, 4). MLIF data in combination of *p16*^*Ink4a*^ furthermore evidenced more than 60% mtG4^+^td^+^ PPM in aged periosteum were *p16*^*Ink4a*+^ senescent cells, which was much higher in comparison to that of young periosteum (Fig. 2cc3, 5). Moreover, tracing data (Fig. 2dd1) demonstrated that at d14 post-fracture, the descendant lineage of PPM at d5 continuously harbored much increased mtG4 level in aged periosteum (Fig. 2dd2, 4), and meanwhile these mtG4^+^td^+^ PPM were more *p16*^*Ink4a*+^ senescent cells when compared with that in young periosteum (Fig. 2dd3, 5). These data together clearly demonstrate that mtG4 in PPM positively links with aging-associated poor skeletal repair.

### In vivo induction of mtG4 in PPM causes poor repair

**Fig. 3:**
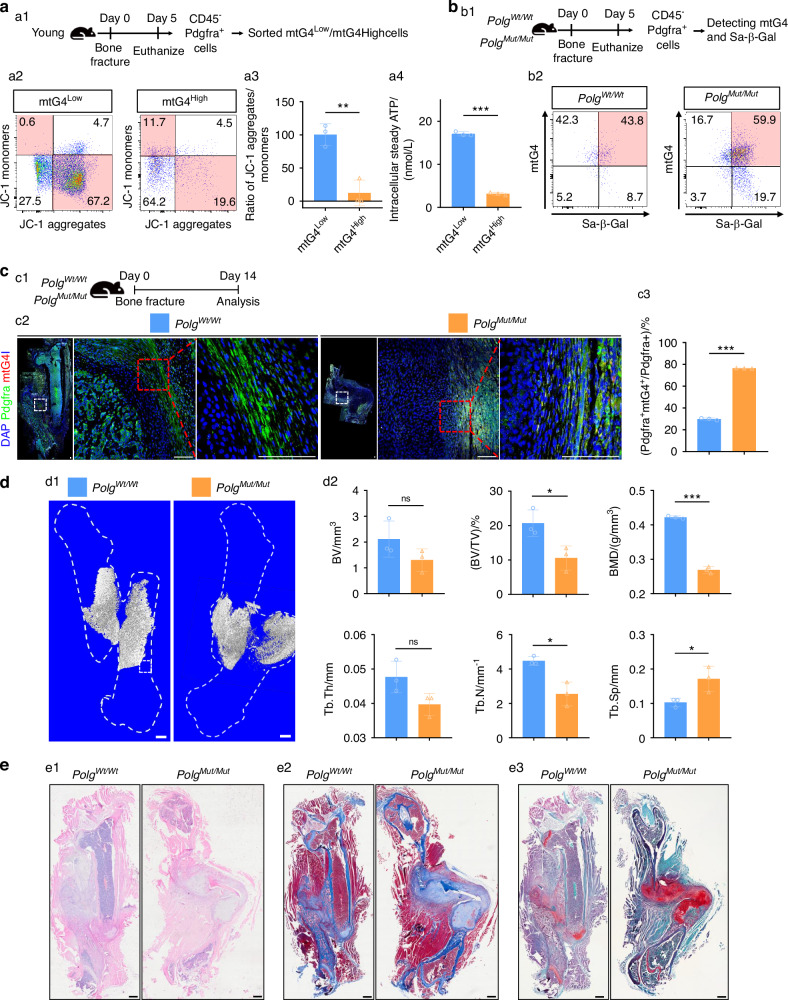
In vivo induction of mtG4 in PPM causes poor skeletal repair. **a** (a1) Schematic of this experiment. (a2) Representative FCM results using JC-1 probes, (a3) quantification results of the ratio of JC-1 aggregates versus monomers, and (a4) the intracellular steady ATP amount between mtG4^Low^ and mtG4^High^ PPM. **b** (b2) Representative FCM results showing the dual-labeling with mtG4 and Sa-β-Gal of PPM within early callus in premature progeria mice, and the schematic of this experiment (b1). **c** (c1) Schematic of the following experiments (**c**–**e**). (c2) Representative MLIF images showing the in vivo induction of mtG4 in PPM at d14 post-fracture. (c3) Statistical data for (c2). Scale bar, 100 μm. **d** (d1) Three-dimensional reconstruction of μCT. Scale bar, 500 μm. (d2) Statistical data for μCT. **e** Representative histopathological staining images of bone callus. (e1) H&E. (e2) Masson’s trichrome. (e3) Safranin O. Scale bar, 500 μm. All experiments were technically replicated three times; *n* = 3 per group. ns no statistical significance. *P < 0.05, **P < 0.01, and ***P < 0.001

After knowing of the positive relation between mtG4 accumulation in PPM and aging-associated poor repair, we next transgenically increased mtG4 in vivo, by using the previously reported in vivo induction model,^24^ to ascertain the effect of mtG4 in bone repair. First, we sorted the CD45^-^ PPM at d5 post-fracture into two groups, which were mtG4^low^ and mtG4^high^ by using FCM (Fig. 3aa1). Next, we incubated these cells using JC-1 probes and then FCM data demonstrated even in young mice the mtG4^high^ PPM owning much less JC-1 aggregates (Fig. 3aa2). Quantification results additionally confirmed the significantly reduced mitochondrial membrane potential in mtG4^high^ PPM when compared with mtG4^low^ population even in young mice (Fig. 3aa3). Intracellular steady ATP level also indicated damaged mitochondrial anabolic functions in mtG4^high^ PPM (Fig. 3aa4). These results implied the harmful effects of mtG4 in PPM’s mitochondrial function. Next, we assessed the in vivo osteogenic capacity of PPM with different mtG4 level (Fig. S4A). Masson’s trichrome and IF results (Fig. S4B b1) demonstrated that mtG4^Low^ PPM regenerated more mineralized tissue (Fig. S4B b2) and OCN-positive tissue (Fig. S4B b3) relative to mtG4^high^ PPM, indicating that mtG4 accumulation functionally impairs PPM-mediated osteogenesis. Therefore, we further verified the consequences of mtG4 accumulation in vivo using *Polg* mutation progeria mice model (homozygous mutations of DNA polymerase gamma, PolgD257A). This well-established progeria model has known mtDNA deletions and damages^25–27^), and our prior study have proven that this mitochondrial dysfunction is related to mtG4 accumulation.^24^ Therefore, we next employed *Polg*^*Mut/Mut*^ to verify the impact of mtG4 on PPM senescence in vivo. FCM data showed that *Polg*^*Mut/Mut*^ PPM owned increased mtG4 level and those mtG4^high^ PPM in homozygous mutants were more Sa-β-Gal^+^ senescent in comparison to that in wild-type (*Polg*^*Wt/Wt*^) littermate controls (Fig. 3bb2). Finally, MLIF data also verified the in vivo induction of mtG4 in PPM (Fig. 3c).

After we affirmed the feasibility and reliability of in vivo model, next we assessed whether mtG4 in PPM affected bone repair (Fig. 3d, e). 3D-RµCT data showed substantially reduced and more porous new bone in *Polg*^*Mut/Mut*^ when compared with control group (Fig. 3dd1). Data of BV, BV/TV, BMD, Tb.Th, and Tb.N. furthermore proved the significantly decreased new bone in mutant group, and meanwhile the up-regulated Tb.Sp indicated more porous structure of the indicated regenerated bone in *Polg*^*Mut/Mut*^ mice (Fig. 3dd2). H&E (Fig. 3ee1), Masson’s trichrome (Fig. 3Ee2), and Safranin O (Fig. 3Ee3) results all showed that in vivo induction of mtG4 in PPM completely phenocopied the aging-associated poor skeletal repair (Fig. S2), which detailedly incudes in reduced mineralized new bone but with increased cartilage tissues in the hard callus. Biomechanical analysis results demonstrated that in vivo induction of mtG4 led to poor load-bearing function (Fig. S3C). These results together proved that in vivo induction of mtG4 in PPM causes aging-like poor bone repair.

### mtG4 drives PPM senescence and poor repair-associated degeneration

**Fig. 4:**
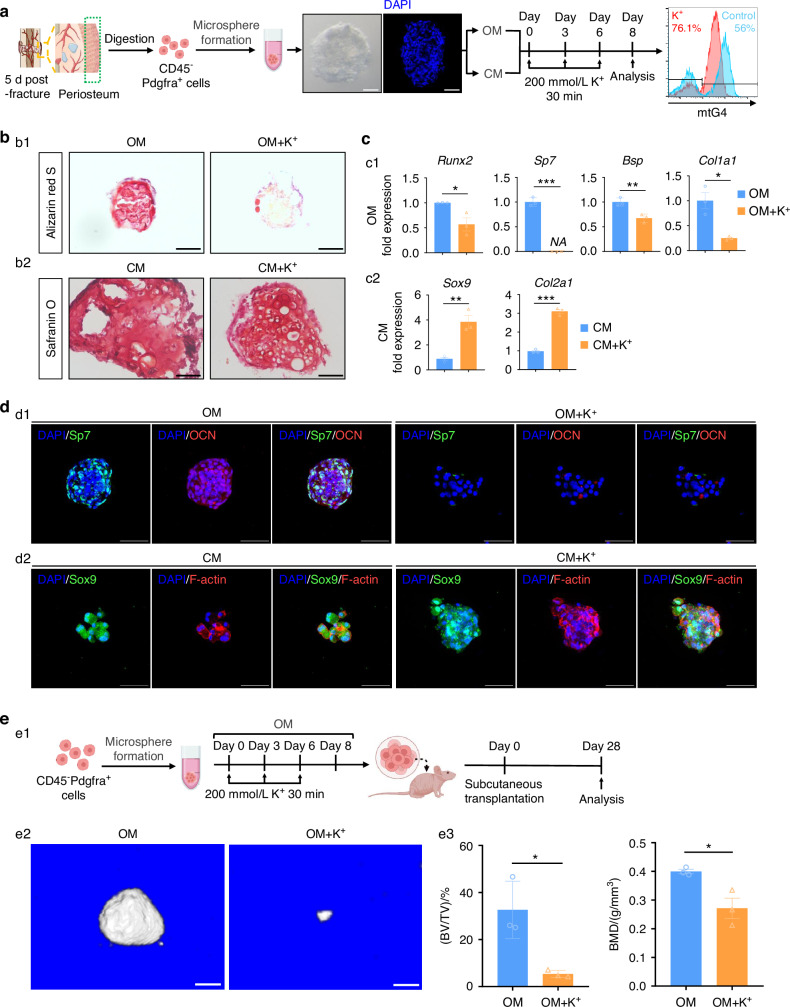
mtG4 drives PPM senescence and poor repair-associated degeneration. **a** Schematic of the following experiments (**b**–**d**) and representative FCM results showing the in vitro mtG4 induction after K^+^ treatment. Scale bar, 50 μm. **b** (b1) Representative images of Alizarin Red S staining. (b2) Representative images of Safranin O staining. Scale bar, 50 μm. **c** RT-qPCR data of (c1) osteogenic genes and (c2) chondrogenic genes in PPM organoids. **d** (d1) Representative MLIF images showing the expression of Sp7 and OCN in PPM organoids. (d2) Representative MLIF images showing the expression of Sox9 and the cell morphology in PPM organoids. Scale bar, 50 μm. **e** (e1) Schematic of this experiment. (e2) Three-dimensional reconstruction of μCT. Scale bar, 100 μm. (e3) Statistical data for μCT. All experiments were technically replicated three times; *n* = 3 per group. ns no statistical significance. *P < 0.05, **P < 0.01, and ***P < 0.001

To investigate the underlying mechanisms by which mtG4 drives PPM senescence and -associated poor bone repair, we carried out comprehensive in vitro assessments by using the CD45^-^ PPM organoids derived from the MSCs infiltration’s periosteum (Fig. S5B). First, we verified the success of establishing early callus-derived PPM organoids (Fig. 4a). Furthermore, following our previously reported in vitro induction model we also successfully induced mtG4 in these PPM organoids by transient and sequential additions of K^+^ (Fig. 4a). Mitochondrial function remains stable under the transient K^+^ stimulation (Fig. S5A). FCM data of Sa-β-Gal next proved these mtG4^high^ PPM’s organoid MSCs, which were induced by K^+^, demonstrated significantly increased senescent proportion (Fig. S5B, C). These data provided direct evidence that inducing mtG4 caused PPM senescence in vitro. After the success of model establishment, we next respectively carried out the osteogenic induction (by using osteogenic conditional medium, OM) and chondrogenic induction (by using chondrogenic conditional medium, CM) of PPM organoids (Fig. 4a). After 8 d’s osteogenic induction we found that the K^+^ group showing substantially reduced mineralization capabilities when compared with the control group (Fig. 4Bb1). RT-qPCR data of vital osteogenic genes, such as *Runx2*, *Sp7*, *Bsp*, and *Col1a1*, consistently demonstrated the impaired osteogenesis in K^+^ treated PPM organoids (Fig. 4c). On the contrary, Safranin O data showed K^+^ treated PPM organoids owning significantly stronger chondrogenesis with much more cartilage extracellular matrices and increased hypertrophy of chondrocyte-like cells (Fig. 4bb2). The vital chondrogenic genes of Sox9 and Col2a1 furthermore proved the enhancement of chondrogenesis in K^+^ group (Fig. 4c). 3D-MLIF data detailly showed after 8 d’s osteogenic induction the PPM organoids were much smaller and owned substantially reduced Sp7^+^ and OCN^+^ proportions in K^+^ treatment group (Fig. 4dd1). But when it came into the chondrogenic induction; however, K^+^ treated PPM organoids showed much more mature hypertrophy, larger tissue size, and increased Sox9^+^ cells (Fig. 4dd2). These data clearly revealed that induction of mtG4 strongly disturbed the fate determination of PPM by inhibiting osteogenic but harnessing chondrogenic commitment, which explained the underlying reasons of in vivo induction of mtG4 causing aging-like poor bone repair, whose typical alterations including decreased osteogenesis but enhanced cartilage formation (Fig. 3).

To finally confirm the role of PPM’s mtG4 in bone regeneration, we subcutaneously transplanted the osteogenic committed PPM organoids into adult null mice (Fig. 4ee1). 3D-RµCT showed the mineralized bone mass was severely decreased in K^+^ group when compared with that in the control group (Fig. 4ee2). Quantification of BV/TV and BMD furthermore proved the impaired bone regeneration of these mtG4-accumulated PPM organoids.

### Mitochondrial dysfunction underlies mtG4-caused PPM senescence

**Fig. 5:**
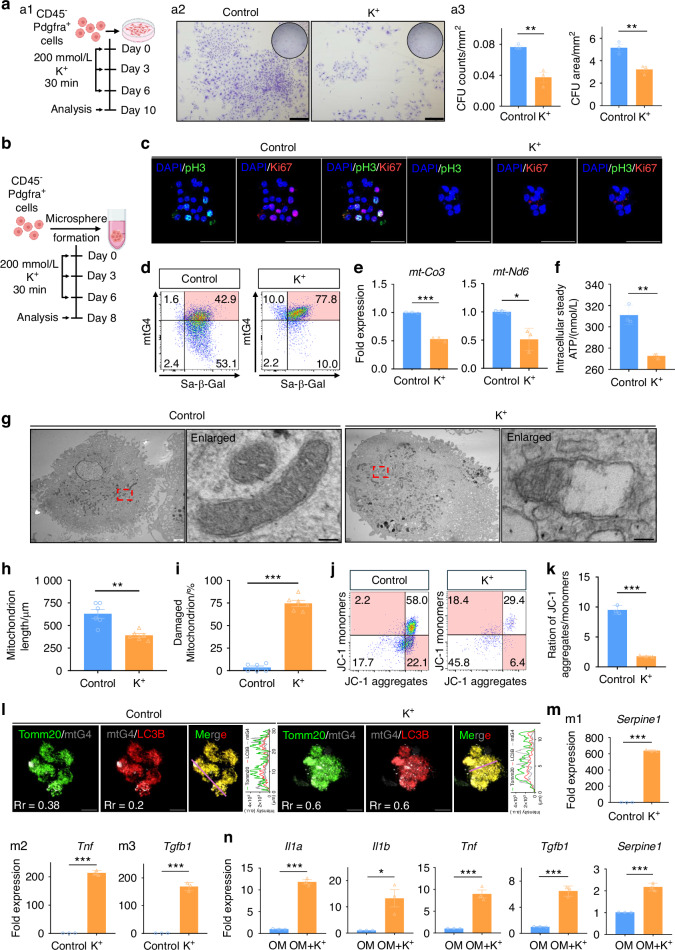
Mitochondrion dysfunction underlines mtG4-caused PPM senescence. **a** (a1) Schematic of this experiment. (a2) Representative images of CFUs. Scale bar, 1 mm. (a3) Statistical data for (a2). **b** Schematic of the following experiments (**c**–**m**). **c** Representative MLIF images showing the expression of Ki67 and pH3 in PPM. Scale bar, 50 μm. **d** FCM results showing the dual-labeling with mtG4 and Sa-β-Gal of PPM. **e** RT-qPCR data of mitochondrial genes in PPM. **f** The intracellular steady ATP amount of PPM. **g**–**i** (**g**) Representative TEM images of PPM and mitochondria. **h**, **i** Statistical data for (**g**). Red dashes outline the enlarged area in the left image. Scale bar, 2 μm. Scale bar for enlarged images, 100 nm. **j**, **k** Representative results of FCM using JC-1 probes and statistic data of the ratio of JC-1 aggregates versus monomers. **l** Representative MLIF images showing the co-labeling of Tomm20, mtG4, and LC3B. The Pearson’s coefficients were all listed in the images. Scale bar, 10 μm. **m** RT-qPCR data of senescence-associated secretory phenotype genes in PPM. **n** RT-qPCR data of senescence-associated secretory phenotype genes in PPM following the experimental procedure in Fig. 4a. All experiments were technically replicated three times; *n* = 3 per group except *n* = 6 in (**h**, **i**). ns no statistical significance. *P < 0.05, **P < 0.01, and ***P < 0.001

After revealing the functional roles of mtG4 in aging-associated poor bone repair, we next investigated the underlying mechanisms. First, we demonstrated that K^+^ itself does not impair mitochondrial function (Fig. S5A). Next, we carried out the self-renewal assessments of 2D-cultured PPM (Fig. 5aa1), and results demonstrated that mtG4 induction group showed substantially reduced colony formation units (CFU) (Fig. 5aa2). Both CFU numbers and areas were significantly down-regulated in the mtG4 induction group (Fig. 5aa3). We verified these findings in 3D PPM organoids (Fig. 5b). MLIF data of PPM organoids showed that mtG4 induction group formed much smaller sphere when compared with the control group (Fig. 5c). Besides, the mitotic hallmark of S-G_2_-M, namely phosphorylated histone H3 (pH3), and the general proliferation marker Ki67 were both barely detected in the mtG4 induced PPM organoids (Fig. 5c). Next, we digested these PPM organoids into single cell suspension which were then subjected into FCM analyses (Fig. 5d). FCM data showed that K^+^ treatment significantly increased mtG4 contents in PPM organoids, and meanwhile these mtG4^high^ organoid cells in induction group became Sa-β-Gal^+^ senescent cells (Fig. 5d). RT-qPCR of PPM organoids showed that in the mtG4 induction group, the previously known mtG4 sensitive mitochondrial genes (*mt-Co3* and *mt-Nd6*)^24^ were significantly down-regulated (Fig. 5e), along with the substantially decreased intracellular steady ATP (Fig. 5f). These data indicated severe mitochondrial dysfunction in mtG4 induced PPM organoids, and provided that mtG4 inhibited the transcription of vital mitochondrial respiratory chain genes to impair mitochondrial anabolism.

Thus, we harvested the PPM organoids and carried out transmission electron microscopic measurements (TEM). TEM data demonstrated that in the mtG4 induction group the cell morphology of PPM was normal in comparison to that in the control (Fig. 5g). And mtG4 neither change the vital membrane organelles such as plasma membrane and nuclear membrane (Fig. S5C c3). However, mtG4 induction specifically caused severe damage in mitochondria (Figs. 5g, S5Cc3). In detail, in the mtG4 induction group mitochondria become much rounder and swelling, and the rarefaction or destruction of mitochondrial crista emerged, showing typical mitophagy appearances (Fig. 5g, S5C c3). Quantitative results furthermore demonstrated the decreased mitochondrial length (Fig. 5h), increased numbers of damaged mitochondria (Fig. 5i), and significantly exacerbated mitophagy of organoids’ PPM in the mtG4 induction group (Fig. S5C c4). These results corroborated the pathological mitochondrial morphology alterations observed in PPM during healthy aging (Fig. S6A). When we returned to the in vivo PPM at the stem cell infiltration stage after fracture, we also observed much more shortened dotted mitochondria in the mtG4-accumulated PPM (Fig. S6B) and whose lineage descendants in the bone callus (Fig. S6C). In vivo induction of mtG4 further showed that *Polg* mutant PPM in the early callus consistently demonstrated more shortened dotted mitochondria (Fig. S6D). Finally, we detected the mitochondrial functions and JC-1 data showed that K^+^-treated PPM organoids owning significantly reduced mitochondrial membrane potential (Fig. 5j, k). Direct mitophagy detections by using LC3B adenoviruses and MLIF (mtG4 and Tomm20) demonstrated that the PPM of K^+^-treated organoids having significantly exacerbated mitophagy (Fig. 5l). In vivo MLIF also verified that both in healthy aging and premature aging these mtG4-accumulated PPM after bone fracture showed substantially increased mitophagy (Fig. S7). At last, we observed that these mtG4-accumulated PPM organoids demonstrated hundreds of times up-regulated senescence-associated secreting phenotype (SASP) genes such as *Serpine 1* (Fig. 5mm1), *Tnf* (Fig. 5mm2), and *Tgfb1* (Fig. 5mm3), and pro-inflammatory cytokines such as *Il-1b* and *Il-6* (Fig. S5Cc5). And even during the osteogenic induction these SASP genes were consistently higher in the mtG4 induction group than that in the control group (Fig. 5n). These results together comprehensively establish that mitochondrial dysfunction underlies the mtG4-caused PPM senescence.

## Discussion

Recent advances on single-cell transcriptomics and transgenic lineage tracking of *p16*^*Ink4a*+^ cells in multiple murine organs have demonstrated both the common characteristics and tissue specificities of aging.^28,29^ These breakthroughs yet have not been included in skeletal system.^28,29^ Thus, up to date the periosteum senescence in aging-associated poor skeletal repair remains unacknowledged.^5,6^ In this study, we reveal mtG4 accumulation in PPM controlling periosteum senescence-caused poor repair. Due to the progression of osteoporosis in aging, fragility fracture, fall, and other types of skeletal injuries substantially threaten the elders through significantly increasing the risks of disability and even death.^1–4^ Poor repair after fracture is the reason.^1–4^ Skeletal stem cell senescence and its inflammatory and degenerative environments have been reported to be involved in aging-associated poor skeletal repair.^7–9^ Most currently known mechanisms of this issue are focused on nuclear genome and -mediated molecular functions.^7–9^ Although mitochondrial dysfunction has already been evidenced as the ubiquitous hallmark of senescence, we have very limited knowledge on how mitochondrial dysfunction is driven, which types of bone cells are sensitive to mitochondrial dysfunction, and the exact pathological roles of mitochondrial dysfunctions in aging-associated poor skeletal repair.^7–9^ To this end, our data respectively answered that mtG4 in PPM drives mitochondrial dysfunction, specifically in periosteum, to pathologically cause disturbed fate determination and degenerative alterations of skeletal stem cells, which finally leads to poor skeletal repair in aging.

The most phenotypic degeneration of insufficient skeletal repair during aging is the disturbed fate determination of bone MSCs,^7–9^ which is demonstrated as inhibited osteogenesis but enhanced chondrogenesis together, finally causing increased cartilage tissues existing within bone callus with reduced mineralized new bone.^10^ Our data reveal the mitochondrial genome reasons underlying this type of degeneration. Although PDGFRα is known to broadly mark mixed cell populations in various tissues, recent research has confirmed that Pdgfra^+^ cells within the periosteum are a distinct population of MSCs.^12,30^ Via using in vivo transgenic and in vitro chemical inductions, we discover that increasing mtG4 directly impairs the self-renewal and osteogenic commitment of PPM, but substantially promotes its chondrogenic formation. It is of note that the up-regulated SASP is observed both during osteogenic and chondrogenic after mtG4 induction, which implies the inhibitory but positive roles of SASP in controlling the respective osteogenesis and chondrogenesis. We finally proved that the PPM organoids with increased mtG4 failed to regenerate mineralized bone after ex vivo transplantation.

To ensure the robustness of these findings while strictly adhering to ethical reduction principles, we implemented rigorous validation strategies. Given the scarcity of primary PPMs, we validated the safety profile of the K^+^ induction protocol in rat BMSCs, a surrogate justified by the high evolutionary conservation of mitochondrial function and the biophysical universality of mtG4 dynamics across different species.^24,31,32^ Besides, our in vivo study adopted a stratified experimental design to balance statistical robustness with ethical reduction principles; fundamental characterizations of mtG4 kinetics in early callus utilized larger cohorts to rigorously overcome biological variability, establishing substantial effect sizes that justified the use of focused groups for subsequent mechanistic testing, which were further substantiated by convergent in vitro and ex vivo data.

In addition to these phenotypic findings, we furthermore reveal the underlying mechanisms of impaired skeletal repair. In detail, our data show that mtG4 impair the transcription of vital mitochondrial genes, including *mt-Co3* and *mt-Nd6*, further breaking the respiratory chain function, to ultimately damage both structural and functional integration of mitochondria in PPM. This finding is consistent with our results discovered in human and murine dental MSCs,^24^ together proving that mtG4 regulates mitochondrial genes transcription to regulate mitochondrial function. These data comprehensively unravel the effects of mtG4 on contributing to aging-associated mitochondrial dysfunctions.^33^

Although we employed K^+^ treatment to experimentally modulate mtG4 levels, we rigorously excluded non-specific off-target effects through multiple controls. As established in our previous work, treatment with equimolar Na^+^ failed to recapitulate the mitochondrial defects observed with K^+^, effectively ruling out osmotic factors.^24^ This distinction stems from the lower hydration free energy of K^+^, which facilitates its dehydration and subsequent coordination within the central cavity of the G-quadruplex, thereby stabilizing the structure via electrostatic interactions.^34^ Crucially, our pulse-and-washout protocol ensured the restoration of cellular ionic homeostasis, verified by unaltered mitochondrial membrane potential and respiratory chain complex activity,^24^ thereby decoupling the structural effects from transient ionic stimulation. Coupled with the observation that transcriptional suppression was strictly gene-specific—restricted to the G4-enriched *mt-Nd6* and *mt-Co3* loci while sparing *mt-Co2*—these data collectively confirm that the observed phenotype is driven by the persistent structural stabilization of mtG4.^24^

Our conclusion that mtG4 suppresses *mt-Nd6* and *mt-Co3* expression represents a biological deduction supported by both sequence-specific biophysical potential and inducible repression data,^24,35,36^ although we acknowledge that further studies are needed to directly capture the physical blockade of transcription. While pharmacological inhibition is often used to probe mitophagy, the known off-target effects of agents like Mdivi-1^37^ on mitochondrial respiration render them unsuitable for our model, which already suffers from transcriptional respiratory defects. We therefore conclude that the bioenergetic crisis driven by mtG4-induced transcriptional blockade is the primary cause of senescence, with mitophagy acting as a secondary, pathological trigger initiated by this irreversible upstream damage.

Broadening this mechanistic view, our findings indicate a deleterious feed-forward loop linking mtDNA damage to mtG4 accumulation. Age-associated replication stress, as recapitulated in the *Polg*^*D257A*^ mouse model, initially primes G4 folding by exposing single-stranded DNA templates. These stabilized mtG4 structures subsequently impede the replication machinery, plausibly consolidating transient errors into permanent genomic instability, thereby accelerating periosteal senescence. Given that other mtDNA mutator models sharing similar mutation burdens do not exhibit the severe bone aging seen in *Polg*^*D257A*^ mice, random mutations cannot account for the observed phenotype.^38^ Our data from wild-type PPMs, where mtG4 induction in vitro recapitulated the aging defects in vivo, identify mtG4 accumulation as the primary determinant of mitochondrial dysfunction and cellular senescence, distinct from the background of global mtDNA mutations. We postulate that the specific accumulation of mtG4 in PPMs stems from the heightened metabolic demands of bone regeneration, which necessitate active mtDNA replication and transcription.^39^ These processes inevitably prolong the exposure of single-stranded mtDNA intermediates, thereby increasing the susceptibility of this critical regenerative population to G-quadruplex formation.^16–18^

By deleting senescent cells in vivo previous transgenic mice data have established that cellular senescence directly contributed to aging,^40^ which spurred the development of senolytic therapies.^40^ The cornerstone of senolytics is to figure out the precise and reliable senescent hallmarks,^40^ but current knowledge has demonstrated that even those “ubiquitous” hallmarks of cell senescence, such as *p16*^*Ink4a*^, still showed organ/cell type heterogeneity and tissue-biased specificity.^28,41–43^ The observed partial, yet significant, overlap between mtG4 positivity and SA-β-Gal activity aligns with the established biological complexity of cellular senescence. These two markers report on distinct but interconnected aspects of the senescent state: mtG4 signifies a specific genomic structural alteration within mitochondrial DNA,^24^ whereas SA-β-Gal activity reflects a subsequent, metabolically active lysosomal phenotype.^44,45^ The presence of mtG4⁺ SA-β-Gal⁻ cells likely represent an early phase where mitochondrial genomic stress precedes full lysosomal activation, while SA-β-Gal⁺mtG4⁻ cells may indicate alternative senescence entry pathways. Critically, the substantial expansion of the double-positive population under aging and fracture stress underscores a strong functional link, positioning mtG4 accumulation as a potent upstream driver contributing to the development of a canonical senescent phenotype.

Our data in this study plumb evidence the existence of tissue-specific senescence hallmark again, which a critically in charge of PPM’s repair capability during aging. Theoretically, by developing mtG4-targeted senolytic therapeutics, according to our findings, poor skeletal repair perhaps can be alleviated to resolve this huge healthspan and lifespan challenge during aging.^5,6,24^ Different from Rapamycin, diet management, and other types of longevity therapies, the mode of action of senolytics is to directly clear senescent cells.^40^ During the clearing process, it is very important to precisely locate and target those harmful senile cells without interfering healthy cells.^40^ Therefore, revealment of the tissue-specific and even disease-specific senolytics, instead of currently available methods of non-selective clearance of senescent cells, will improve both the feasibilities, efficacy, success ratio, and safety of senolytic therapies.^40^ To this end, our data show the accumulated mtG4 specifically appearing within the aged periosteum, and the increased mtG4 in periosteum pathologically responsible for poor bone repair. Therefore, our findings theoretically provide the first potent path towards the targeted senolytic therapy specific for aging-associated skeletal repair, which will open the door of developing precise senolytics.

In summary, we report that the special mitochondrial DNA structure, namely mtG4, is significantly increased in both physiological and pathological aging, driving mitochondrial dysfunctions, finally causing PPM senescence to phenocopied aging-associated poor skeletal repair. Our findings shed light on the mitochondrial mechanisms of periosteum senescence in aging-associated poor repair, which provides with the potent target of alleviating insufficient bone repair in elders. Developing mtG4-targeted senolytic or mtG4 resolving agents theoretically endow us with brand-new avenue towards enhancement of bone regeneration to reduce the huge burdens and death risks of fracture in elders.

## Materials and Method

### Mice

All animal experimentation protocols were subjected to thorough review and received approval from the Ethical Committees of the West China School of Stomatology, Sichuan University (Approval No. WCHSIRB-D-2022-201). The corresponding surgical models were implemented in accordance with the established guidelines of the State Key Laboratory of Oral Diseases at West China Hospital of Stomatology. With respect to the maintaining of experimental mice, they were housed in specific-pathogen free Experimental Animal Core of West China Hospital, Sichuan University (China), which was a temperature controlled (25 °C) environment under a 12-h light/12-h dark cycle with cotton batting.

*Pdgfra-CreER* (Stock#018280), *tdTomato* (Stock#007914), and *Polg*^*D257A*^ (stock#017341) were purchased from JAX lab. To get the homozygous mutant of *Polg*^*D257A*^, we followed the guidelines from JAX lab. In brief, the female and male heterozygous *Polg*^*D257A*^ mice (that originated from a C57BL/6 J female x heterozygous male cross) were crossed to get the homogenous strain. The primer design of *Polg*^*D257A*^ mice was shown in Table S1, and the gene identification was shown in Figure S8.

### In vivo mouse studies

For tamoxifen (TAM) administration, mice were intraperitoneally injected with corn oil-diluted TAM with the concentration of 70 mg/kg weight.

### Femoral bone regeneration

The bone regeneration model was reported by us previously.^25^ In brief, in all animal surgical procedures, inhalation anesthesia with isoflurane (typically 1.5%–3% in oxygen) was used for induction and maintenance. buprenorphine was given 60 min prior to surgery. One of the legs was shaved and scrubbed with 75% ethanol. Closed, nonstabilized fractures were generated by three-point bending. The mice were euthanized at d3, d5, d7, and d14 post-fracture for further analysis.

### Isolation of mouse Pdgfra^+^ periosteal MSCs

For Pdgfra^+^ periosteal MSCs isolation, d5 post-fracture, the fibrous callus component in early callus was separated and cut into pieces. Then, fibrous callus was digested in 1 mL pre-warmed digestion solution (3 mg/mL collagenase I (Sigma, USA) in αMEM) for 45 min in a water bath at 37 °C with gentle agitation every 10 min. Then, the mixture of digested minced bones and target cells were treated with red blood cell lysate on ice for 3 min. After lysis, single-cell suspension was sorted using CD45 MicroBeads (Milteny, #130-052-301, Germany) and CD104a (Pdgfra) MicroBeads Kit (Milteny, #130-101-502, Germany). Enriched CD45^-^Pdgfra^+^ periosteal MSCs were collected for further experiments.

For flowcytometry analysis of periosteum at homeostasis, the femur was cut at 1 mm away from the femoral head and another cut at 1 mm away from the femoral condyle to obtain the bone shaft. Bone marrow and endosteal cells were flushed out with cold PBS through a 1 mL syringe needle. The remaining bone shaft was cut into 2 halves along the longitudinal axis. After removing the residue of endosteum, bone pieces were digested in 1 mL pre-warmed digestion solution (3 mg/mL collagenase I (Sigma, USA) in αMEM) for 45 min in a water bath at 37 °C with gentle agitation every 10 min. The subsequent procedures were the same as above.

### Senescence-associated β-galactosidase analysis

Bone tissues were fixed overnight in 4% paraformaldehyde/PBS at 4 °C, followed by decalcification for 7 days in 12% EDTA. After decalcification the tissues were immersed in a 30% sucrose solution overnight at 4 °C. Then samples were embedded in an OCT tissue-freezing medium (Leica) to conduct frozen sectioning at 6 μm thickness using a CryoJane Tape Transfer System (Leica). Frozen sections were treated with 0.1% Triton X-100/PBS (PBST) for 15 min. After blocking w/5% BSA in PBST for 20 min, the cells were detected with Senescence Assay Kit (Beta Galactosidase, Fluorescence) (Abcam, USA), according to the manufacturer’s instructions. Then cells were incubated with TPA-mTO, which was an efficient and reliable mtG4-specific fluorescent probe,^24^ at room temperature for 45 min. Finally, cells were washed using PBS 3 times at 10 min per time, mounted using anti-fluorescence fate mounting medium, and detected by confocal laser scanning microscopy (Olympus, Japan) following the Alexa Fluor 488 protocol for Sa-β-Gal, and Ex@543 nm for TPA-mTO channel (600–700 nm).

As for FCMs analysis, freshly prepared cells were incubated with Senescence Assay Kit (Beta Galactosidase, Fluorescence) (Abcam, USA) for 2 h at 37 °C and TPA-mTO at room temperature for 45 min. Flow cytometric analysis was performed on FACSAria SORP (BD, USA).

### PPM organoids

To form the microsphere organoids of PPM from the fractured sites at d5 post-fracture, procedures were carried out in accordance to a previously published literature^46^ with modifications. Briefly, CD45^-^Pdgfra^+^ cells (1.5 × 10^5^) were culture in 15 mL centrifuge tubes with non-treated surface in culture medium consisting of αMEM, 10% FBS, 1% penicillin/streptomycin for 24 h with gentle agitation every 4 h. Microspheres were used for in vitro cell experiments unless otherwise noted, and carried out the osteogenic induction, osteochondrogenic induction, and colony formation experiments using these microsphere organoids following our previous study.^47^

### Pdgfra^+^ periosteal MSCs transplantation

With respect to Pdgfra^+^ periosteal MSCs transplantation experiments, procedures were carried out in accordance to our previously established protocol^48^ with modifications. Briefly, microsphere organoids were collected and mixed with Matrigel (Corning, USA). Then, microspheres/Matrigel composites were subcutaneously transplanted in null mice. 28 days after transplantation the composites were harvested for further analysis.

### BMSCs cell culture

Male SD rats at 2 weeks were used to obtain initial (P0) bone mesenchymal stem cells (BMSCs) from their long bone marrow. using direct adherence as described previously.^49^ In brief, bone shaft was obtained as before. Subsequently, a disposable aseptic syringe was used to draw antibiotic supplemented αMEM medium and to repeatedly wash the bone marrow cavity to collect cells in a sterile flask. The BMSCs were cultured in 25 cm^2^ flasks (Corning, USA) using αMEM, 10% FBS, 1% penicillin/streptomycin in a controlled environment of 5% CO_2_ at 37 °C. The culture medium was refreshed every 2 days, and subculturing was performed once the cells reached 90% confluence. To examine the effects of transient K^+^ stimulation on mitochondrial function in cells, passage 3 (P3) BMSCs were utilized.

### Induction of mtG4 in vitro

KCl was used to induce the mtG4 as described in our previously established protocol.^24^ Briefly, cells were incubating with K^+^ in PBS at a final concentration of 200 mmol/L for 30 min at 37 °C, followed by fresh PBS washing for 3 times. The control group was treated with PBS without additional K^+^. The procedure was repeated every 3 days.

### Mitochondrial function assays

For TEM observation, microspheres were digested to single-cell suspensions and fixed in 2.5% Glutaraldehyde/PBS (1/5) for 5 min and finally detected via JEOL TEM microscope (#JEM-1400PLUS, Japan). For mitochondrial membrane potential detection, microspheres were digested to single-cell suspensions and detected using Mitochondrial membrane potential assay kit with JC-1 (Beyotime, #C2003S, China). And the flowcytometry was carried out using FACSAria SORP (BD, USA). Intracellular steady ATP amount measurements were carried out using Enhanced ATP Assay Kit (Beyotime, #S0027, China) according to the manufacturer’s instruction, and luminescence was detected via the luminometer of Varioskan Flash (Thermo Scientific, USA).

### Colony Formation Assay

With respect to single-cell suspensions of freshly prepared CD45^−^Pdgfra^+^ cells, 30 000 cells were plated into 6-well plates and maintained in 1 mL of αMEM with 10% FBS and 1% P/S. After 10 days of culture, cells were fixed with 4% paraformaldehyde/PBS and stained with crystal violet (Beyotime, China).

### Osteogenic/chondrogenic differentiation

Osteogenic differentiation was performed using osteogenic conditional medium consisting of αMEM, 5% FBS, 1% penicillin/streptomycin, 10 nmol/L dexamethasone, 10 mmol/L glycerophosphate, 50 μg/mL L-ascorbic acid. The medium was changed every 3 d. For chondrogenic differentiation, microspheres were cultured with chondrogenic conditional medium consisting of αMEM, 10 ng/mL transforming growth factor-β3 (Cloud-Clone, China), 100x ITS (Sigma, USA), 100 μg/mL pyruvate, 40 μg/mL proline, 50 μg/mL L-ascorbic acid (Sigma, USA), and 100 nmol/L dexamethasone (Sigma, USA).

After 8 days of differentiation, the microsphere organoids were collected to perform histomorphological staining, immunofluorescence staining, and detect the expression of osteogenic/chondrogenic marker genes.

### Histomorphological staining of cultured microsphere organoids

Microsphere organoids were fixed for 2 h in 4% paraformaldehyde/PBS at 4 °C. After fixation the tissues were immersed in a 30% sucrose solution overnight at 4 °C. Then samples were embedded in an OCT tissue-freezing medium (Leica) to conduct frozen sectioning at 6 μm thickness. The sections were stained with Alizarin Red S staining (Solarbio, China) and Alcian Blue staining (Solarbio, China). Images were taken with an upright optical microscope (Leica, Germany).

### Immunostaining of cultured microsphere organoids

Microsphere organoids were fixed in 4% paraformaldehyde/PBS for 2 h and then were blocked w/5% BSA in PBST for 20 min, the microsphere organoids were incubated with corresponding first antibodies overnight at 4 °C. At the second day microsphere organoids were washed in fresh PBS three times and then were incubated using specific second antibody, TPA-mTO, with/without DAPI for 2 h at room temperature. Finally, microsphere organoids were washed using PBS for 3 times at 10 min per time, mounted using anti-fluorescence fate mounting medium, and detected.

### RT-qPCR

For RT-qPCR total RNA was extracted using the TRIzol™ (Invitrogen, USA) according to the manufacturer’s protocol. Complementary DNA was synthesized by using the HiScript III RT SuperMix for qPCR (Vazyme, China) in accordance to user manuals. Then quantitative real-time PCR was performed in triplicate by using AceQ Universal SYBR qPCR Master Mix (Vazyme, China) for PCR reactions on an iCycler Real-Time Detection System (BioRad, USA). The relative amount of mRNA was normalized to house-keeping gene Glyceraldehyde-3-phosphate dehydrogenase (Gapdh). Primers used for RT-qPCR are listed in Table S1.

### Micro-CT analysis

With respect to μCT assessment, bone tissues were isolated from euthanized mice, then fixed in 4% paraformaldehyde/PBS and transferred to 70% ethanol for storage. All outcomes were obtained via Scanco Medical μCT 35 system (Scanco Medical AG, Bassersdorf, Switzerland) system. The previously established parameters were used as follows: X-ray intensity, 145 μA; integration time, 200 ms; X-ray tube potential, 55 kVp; and threshold, 220 mg/cm^3^.

### Biomechanical analysis

The collected micro-CT data were imported into Mimics Research 19.0. The three-dimensional model containing trabecular and cortical bone was reconstructed and output into STL format. The model was then imported into 3-matic software for smoothing and refining the mesh, removing discontinuous points, and then generating volumetric and surface meshes. The volumetric mesh was imported into ABAQUS 2021 for finite element analysis. Material parameters, including elastic modulus and Poisson’s ratio, were defined, along with boundary conditions and loads. The analysis was solved to compute the volume fraction of element stress and displacement data.

### Histological staining of bone tissues

Bone tissues were fixed overnight in 4% paraformaldehyde/PBS at 4 °C, followed by decalcification for 3 weeks in 12% EDTA and paraffin embedded. Histomorphological analysis was performed on 6 μm thick sections.

The sections were stained with Hematoxylin and Eosin (H&E) (Biosharp, China) staining, and images were taken with a microscope (Leica, Germany). Standard protocols were followed for Mason’s Trichrome staining (Solarbio, China), Safranin O staining (Solarbio, China), and Alcian Blue staining (Solarbio, China).

### Immunostaining of bone tissues

For frozen sections, bone tissues were fixed overnight in 4% paraformaldehyde/PBS at 4 °C, followed by decalcification for 7 days in 12% EDTA. After decalcification the tissues were immersed in a 30% sucrose solution overnight at 4 °C. Then samples were embedded in an OCT tissue-freezing medium (Leica) to conduct frozen sectioning at 6 μm thickness. The sections were later treated with PBST as above and then the procedures were same as the immunostaining of cultured microspheres.

As for paraffin sections requiring to be imaged with TPA-mTO and tdTomato together, after rehydrated sections were further subjected to antigen retrieval for 30 min at 95 °C. The sections were later treated as the same as above. tdTomato was labelled by Alexa Fluor 488.

### Cell counting

We used ImageJ software for cell counting. First, we limit the region of interest (ROI) to the target area. Then we count the DAPI^+^ cells within the ROI as the total cell count. Next, mtG4^+^DAPI^+^ cells were counted as mtG4^+^ cells. Calculations were performed using the following formula:

$${{\rm{mtG}}4}^{+}{\rm{cells\; proportion}}=\frac{{{\rm{mtG}}4}^{+}{{\rm{DAPI}}}^{+}{\rm{counts}}}{{{\rm{DAPI}}}^{+}{\rm{counts}}}\times 100 \%$$

### Statistics and image processing

All results were verified via independently triplicated experiments. All graphic illustrations and statistical analyses were obtained using GraphPad Prism software v7.03. For comparison among multiple groups when appropriate one-way ANOVA followed by Tukey post hoc test was applied. With respect to results such as the comparison between the controls and the experimental groups, two-tailed Student’s *t* test was used to determine the significance of difference. A *P*-value lower than 0.05 was considered significant. All numerical data were reported in format of mean ± SEM from at least three or more independent experiments. All fluorescent images were processed with Image Pro Plus 6.0 (Media Cybernetics, Rockville, MD, USA), Imaris v9.9.0 (Oxford Instruments, UK), or ImageJ (ImageJ software v1.51w). FCM images were processed via FlowJo v10.8.1 (FlowJo LLC, USA).

## Supplementary information

Table S1

Figure S1

Figure S2

Figure S3

Figure S4

Figure S5

Figure S6

Figure S7

Figure S8

Supplementary materials

## Data Availability

Any additional information required to reanalyze the data reported in this paper is available from the lead contact upon request.

## Materials availability

All unique/stable reagents generated in this study are available from Prof. Fanyuan Yu (fanyuan_yu@outlook.com) with a completed Materials Transfer Agreement.

## Code availability

Any additional information required to reanalyze the data reported in this paper is available from the lead contact upon request.
