# Supplementary figures and images for: Periosteal mitochondria DNA structures drive aging-associated poor skeletal repair
Source: Bone Res. 2026 Apr 7;14:40. doi: 10.1038/s41413-026-00524-6 (PMC13056912; doi:10.1038/s41413-026-00524-6)

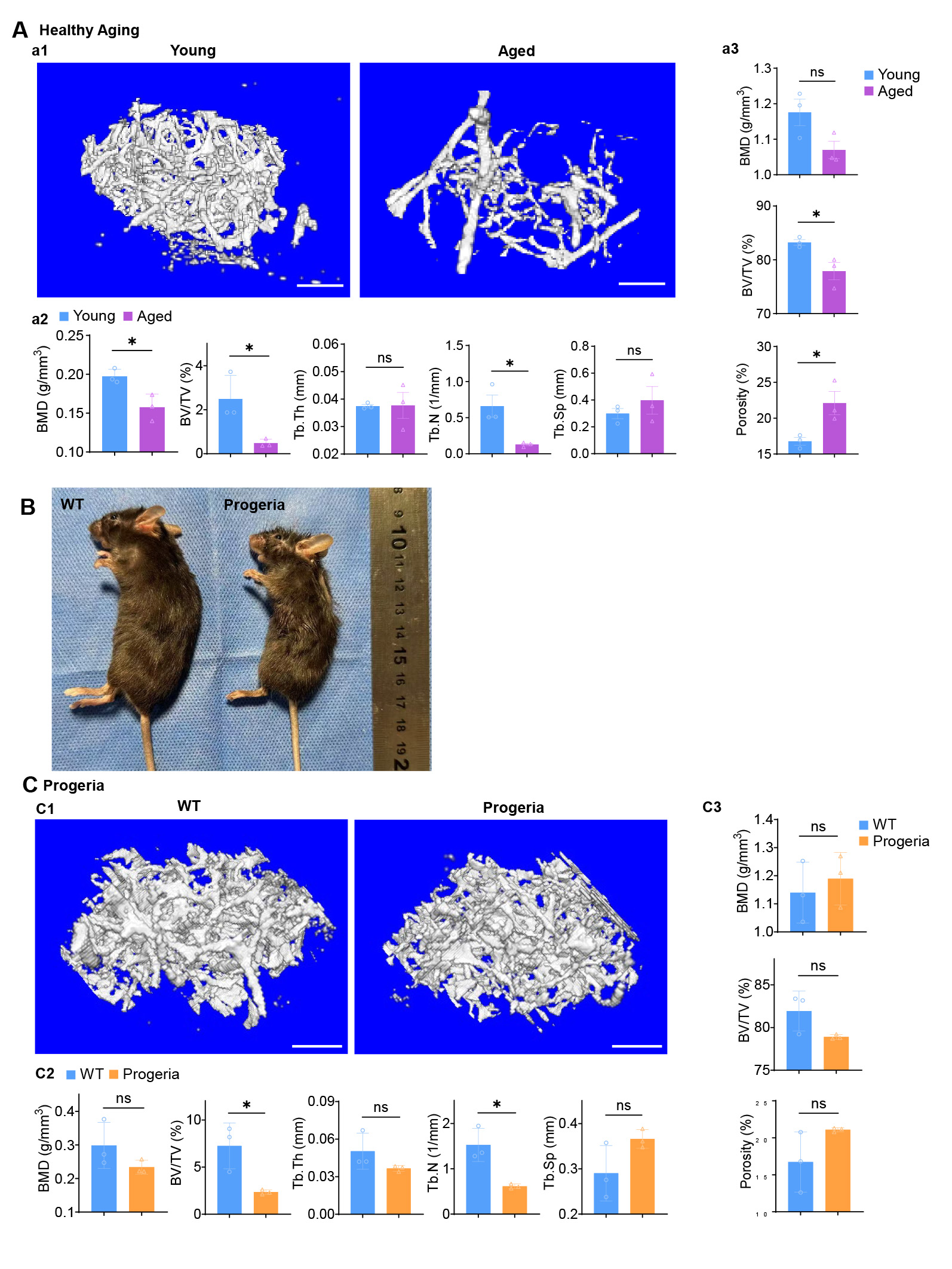

Supplement: Supplementary file 2 — Figure S1 [file 41413_2026_524_MOESM2_ESM.jpg]

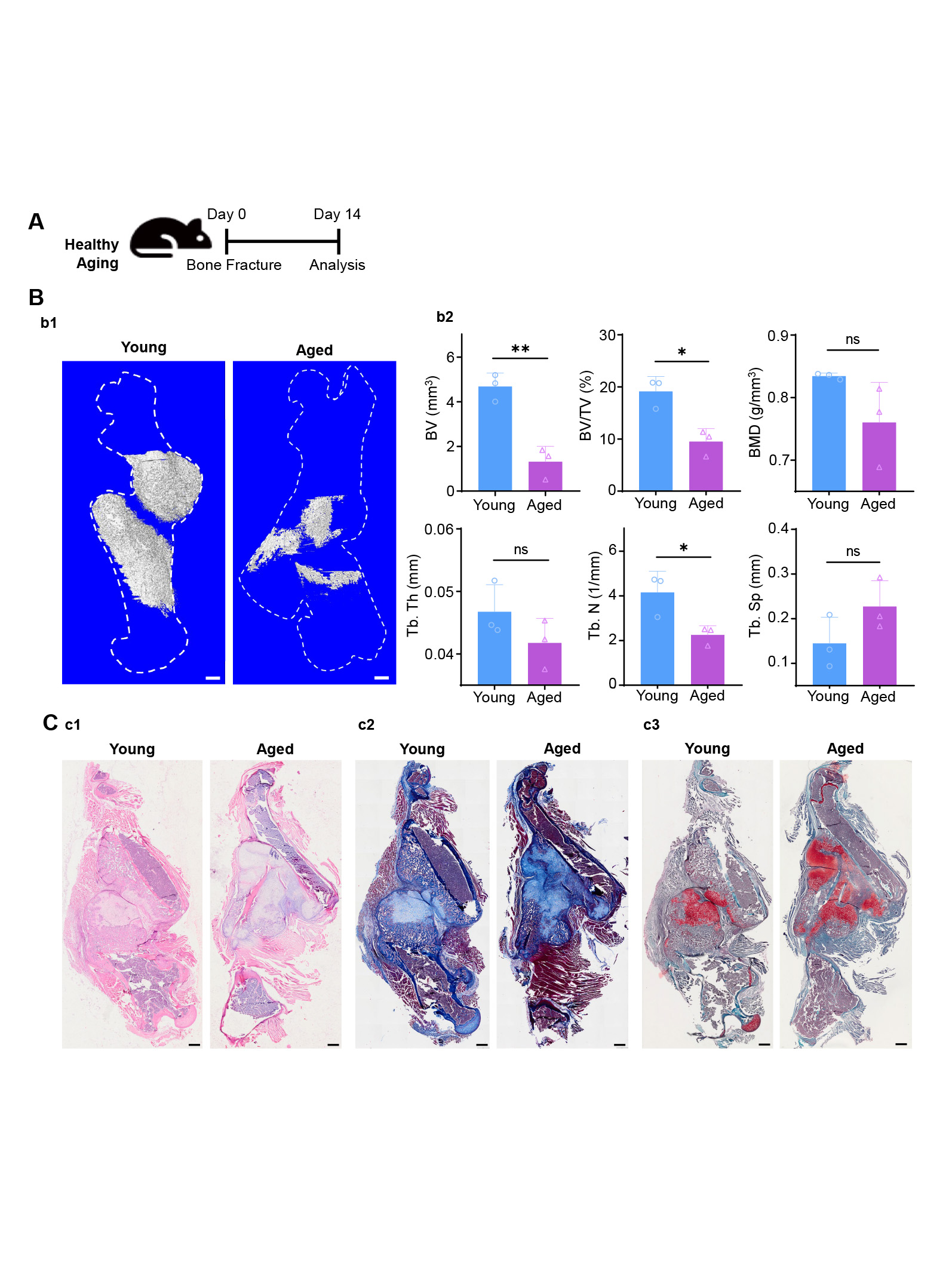

Supplement: Supplementary file 3 — Figure S2 [file 41413_2026_524_MOESM3_ESM.jpg]

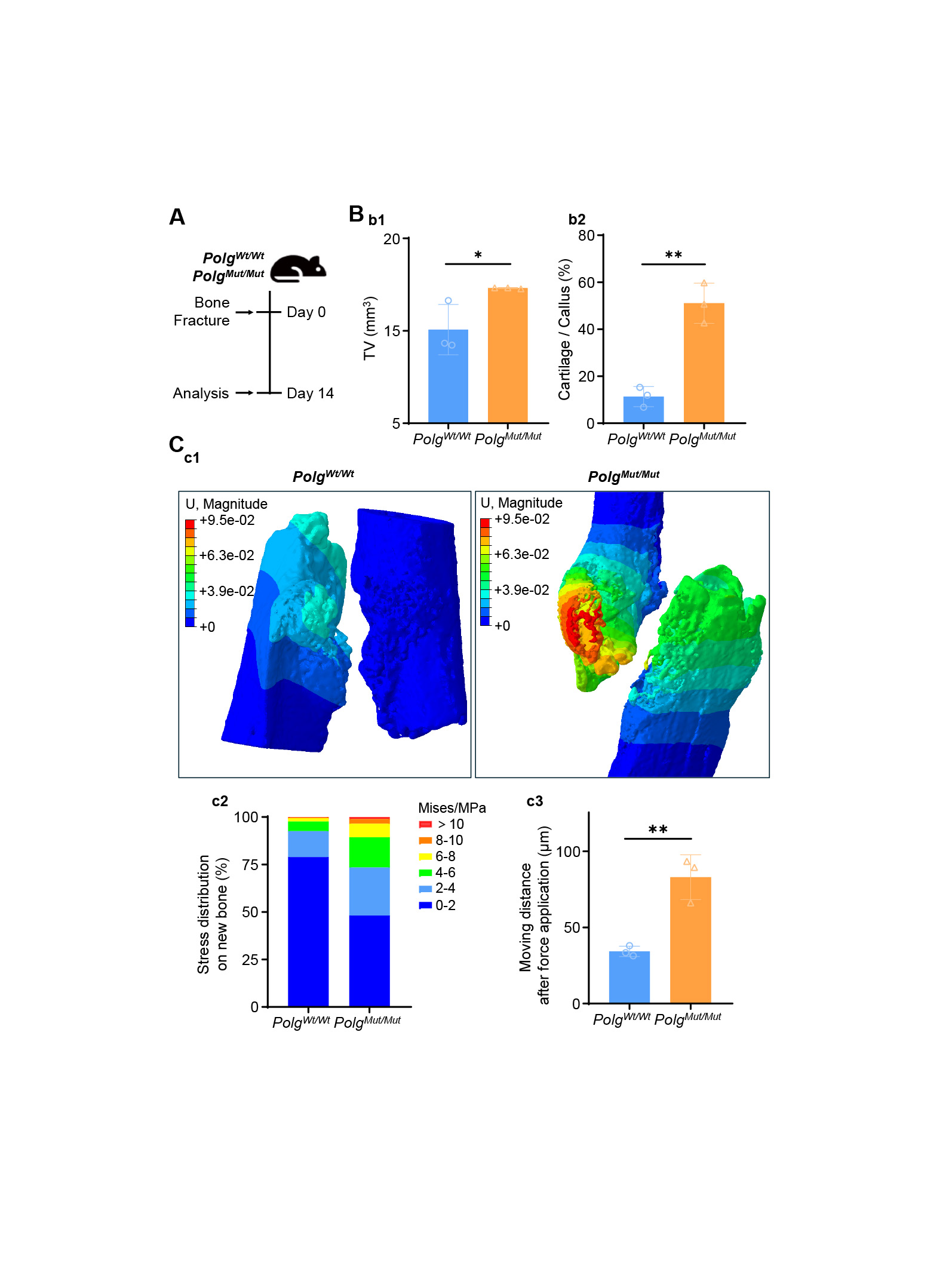

Supplement: Supplementary file 4 — Figure S3 [file 41413_2026_524_MOESM4_ESM.jpg]

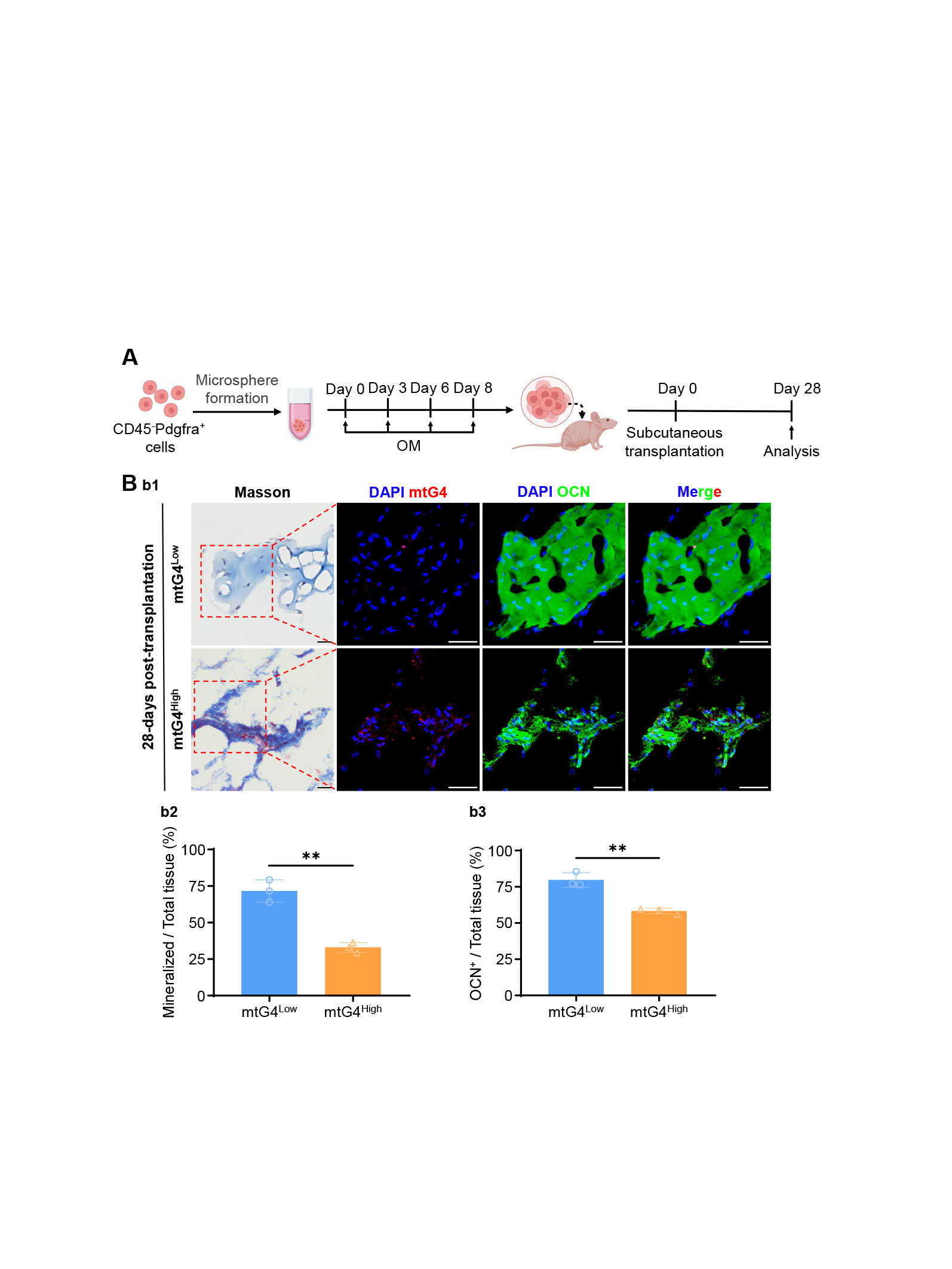

Supplement: Supplementary file 5 — Figure S4 [file 41413_2026_524_MOESM5_ESM.jpg]

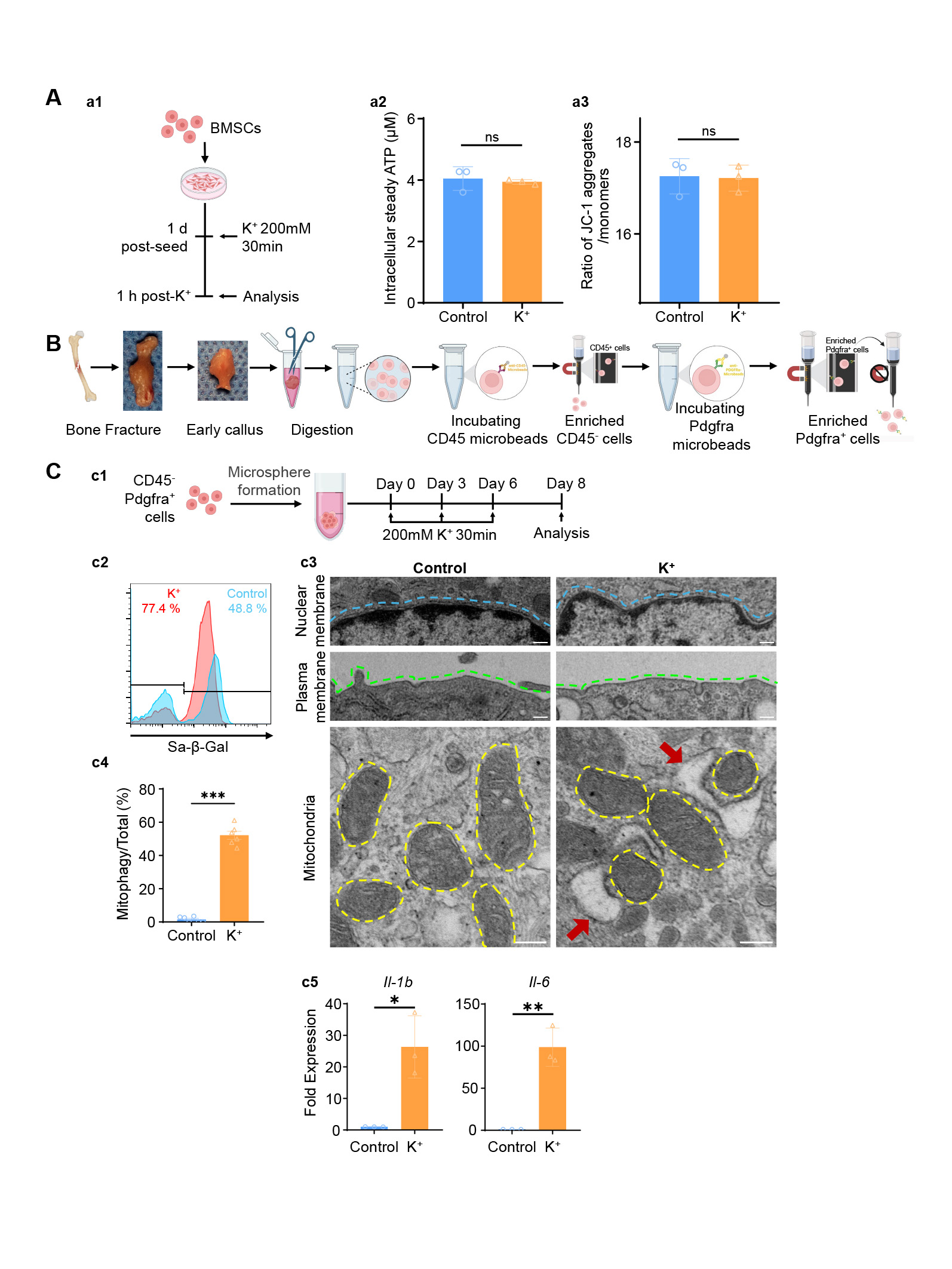

Supplement: Supplementary file 6 — Figure S5 [file 41413_2026_524_MOESM6_ESM.jpg]

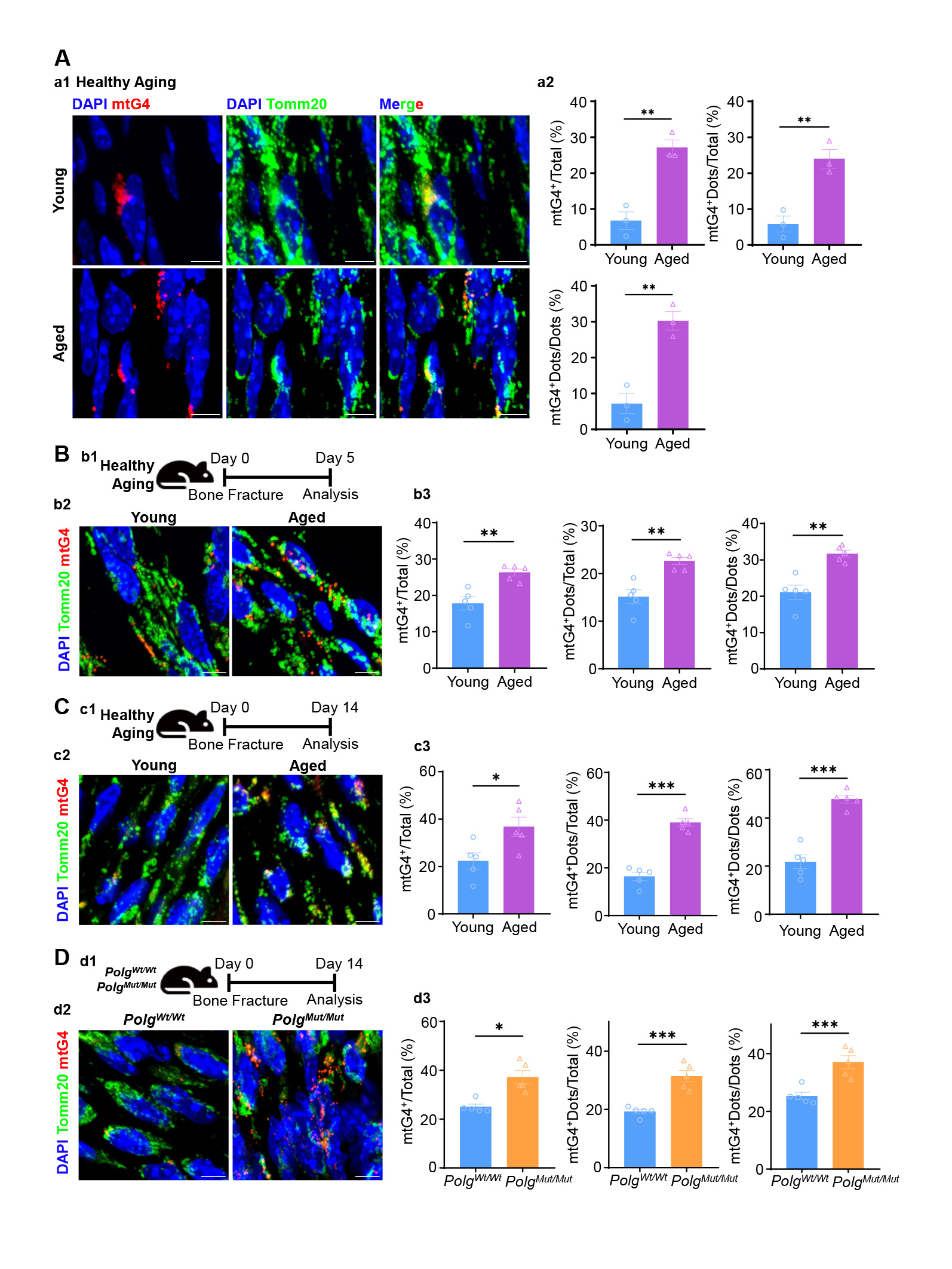

Supplement: Supplementary file 7 — Figure S6 [file 41413_2026_524_MOESM7_ESM.jpg]

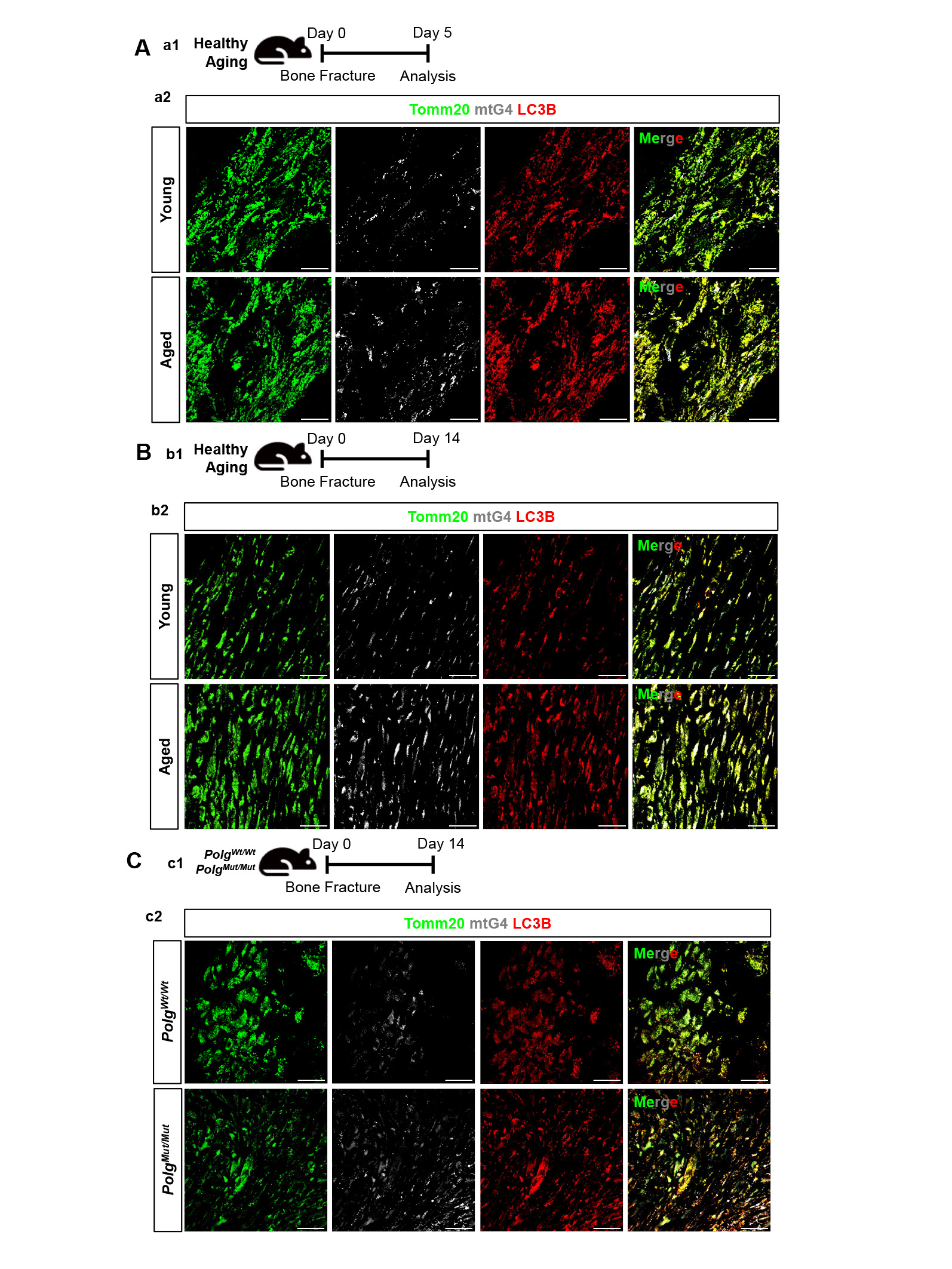

Supplement: Supplementary file 8 — Figure S7 [file 41413_2026_524_MOESM8_ESM.jpg]

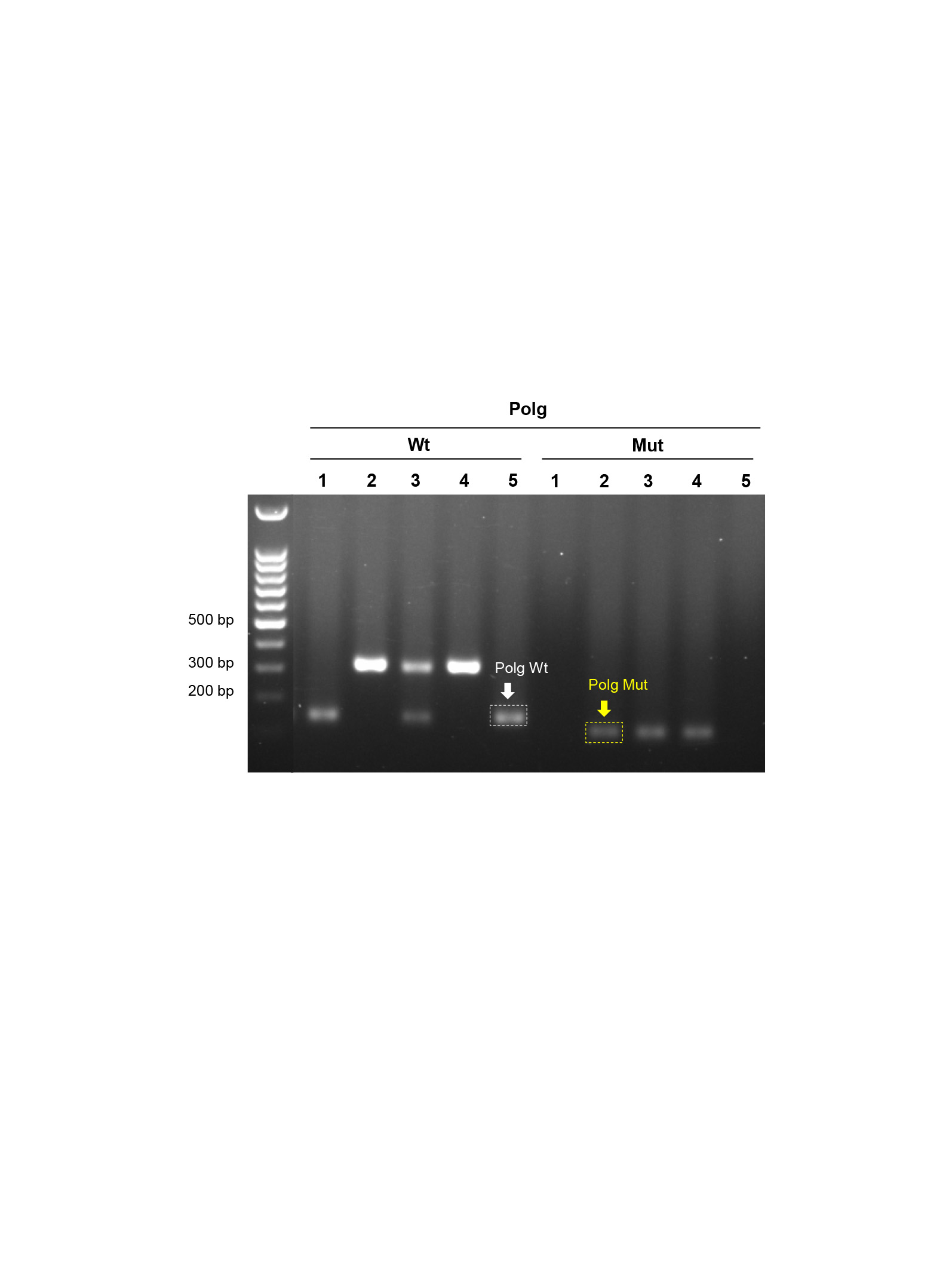

Supplement: Supplementary file 9 — Figure S8 [file 41413_2026_524_MOESM9_ESM.jpg]
