# Supplementary material for: Periosteal mitochondria DNA structures drive aging-associated poor skeletal repair
Source: Bone Res. 2026 Apr 7;14:40. doi: 10.1038/s41413-026-00524-6 (PMC13056912; doi:10.1038/s41413-026-00524-6)
Supplement: Supplementary file 10 — Supplementary materials [file 41413_2026_524_MOESM10_ESM.docx]

**Figure S1. Osteoporosis occurs in both premature and healthy aging.**

(**A**) μCT assay for the femur of healthy aged mice. (a1) Three-dimensional reconstruction of μCT showing the trabecular bones. Scale bar, 200 μm. (a2) Statistical data for μCT of trabecular bones. (a3) Statistical data for μCT of cortical bones. Porosity, total porosity.

(**B**) Optical images of premature progeria mice.

(**C**) μCT assay for the femur of premature progeria mice. (c1) Three-dimensional reconstruction of μCT showing the trabecular bones. Scale bar, 200 μm. (c2) Statistical data for μCT. (c3) Statistical data for μCT of cortical bones.

WT, 5 MO. Progeria, 5 MO; unless stated otherwise. All experiments were technically replicated three times; n = 3 per group. ns, no statistical significance. ∗p < 0.05, ∗∗p < 0.01, and ∗∗∗p < 0.001.

**Figure S2. Aging-associated poor skeletal repair represents an intrinsic phenotype.**

(**A**) Schematic of the following experiments (B-C).

(**B**)(b1) Three-dimensional reconstruction of μCT. Scale bar, 500 μm. (b2) Statistical data for μCT.

(**C**) Representative histopathological staining images of bone callus. (c1) H&E. (c2) Masson’s trichrome. (c3) Safranin O. Scale bar, 500 μm.

All experiments were technically replicated three times; n = 3 per group. ns, no statistical significance. ∗p < 0.05, ∗∗p < 0.01, and ∗∗∗p < 0.001.

**Figure S3. *In vivo* induction of mtG4 causes poor load-bearing function.**

**(A)** Schematic of the following experiments (B-C).

**(B)**(b1) Statistical data for μCT. (b) Statistical data for histopathological staining images.

**(C)**(c1) Representative result of the stress distribution after force application. (c2) Quantitative results of stress distribution on new bone. (c) Moving distance of the callus after force application.

All experiments were technically replicated three times; n = 3 per group. ns, no statistical significance. ∗p < 0.05, ∗∗p < 0.01, and ∗∗∗p < 0.001.

**Figure S4. mtG4 accumulation impairs the osteogenic capacity of PPM.**

**(A)** Schematic of this experiment.

**(B)**(b1) Representative Masson’s trichrome staining and IF images of regenerative tissue derived from PPM. Scale bar, 50 μm. Quantitative statistical results of (b2) mineralized tissue in Masson’s trichrome staining and (b3) OCN^+^ tissue in IF assay.

All experiments were technically replicated three times; n = 3 per group. ns, no statistical significance. ∗p < 0.05, ∗∗p < 0.01, and ∗∗∗p < 0.001.

**Figure S5. *In vitro* induction of mtG4 positively links to mitophagy.**

**(A)**(a1) Schematic of this experiment: impact of K^+^ on mitochondrial function. (a2) The intracellular steady ATP amount. (a3) Statistic data of JC-1 aggregates versus monomers.

**(B)** Schematic of the isolation of mouse PPM.

**(C)**(c1) Schematic of this experiment: induction of mtG4 *in vitro*. (c2) Representative FCM results showing the percentages of senescent PPM after mtG4 induction. (c3) Representative TEM images of PPM after mtG4 induction. Blue dashed lines represent the nuclear membrane. Green dashed lines represent the cell membrane. Yellow dashed circles represent the mitochondria. Red arrows indicate mitophagy. Scale bar, 200 nm. (c4) Statistic data of the percentages of mitophagy. (c5) RT-PCR results of pro-inflammatory cytokines in PPM.

All experiments were technically replicated three times; n = 3 per group except n = 6 in (C). ns, no statistical significance. ∗p < 0.05, ∗∗p < 0.01, and ∗∗∗p < 0.001.

**Figure S6. Accumulation of mtG4 positively links to pathological alterations in mitochondrial morphology.**

**(A)** Pathological alterations in mitochondrial morphology of PPM within periosteum in healthy aging. (a1) Representative MLIF images showing mitochondrial morphology and mtG4 co-labeling. Scale bar, 5 μm. (a2) Statistic data of the percentages of mtG4^+^Dots mitochondria.

(**B**)(b1) Schematic of this experiment: PPM within early callus at d5 post-fracture in healthy aged mice were examined. (b2) Representative MLIF images showing mitochondrial morphology and mtG4 co-labeling. Scale bar, 5 μm. (b3) Statistic data of the percentages of mtG4^+^Dots mitochondria.

(**C**)(c1) Schematic of this experiment: PPM within early callus at d14 post-fracture in healthy aged mice were examined. (c2) Representative MLIF images showing mitochondrial morphology and mtG4 co-labeling. Scale bar, 5 μm. (c3) Statistic data of the percentages of mtG4^+^Dots mitochondria.

(**D**)(d1) Schematic of this experiment: PPM within early callus at d14 post-fracture in premature progeria mice were examined. (d2) Representative MLIF images showing mitochondrial morphology and mtG4 co-labeling. Scale bar, 5 μm. (d3) Statistic data of the percentages of mtG4^+^Dots mitochondria.

All experiments were technically replicated three times; n = 5 per group except n = 3 in (A). ns, no statistical significance. ∗p < 0.05, ∗∗p < 0.01, and ∗∗∗p < 0.001.

**Figure S7. mtG4-accumulated PPM after bone fracture showed substantially increased mitophagy.**

(**A**)(a1) Schematic of this experiment. (a2) Representative MLIF images showing co-labeling of mtG4, Tomm20 and LC3B in PPM within early callus at d5 post-fracture in healthy aged mice.

(**B**)(b1) Schematic of this experiment. (b2) Representative MLIF images showing co-labeling of mtG4, Tomm20 and LC3B in PPM within early callus at d14 post-fracture in healthy aged mice.

(**C**)(c1) Schematic of this experiment. (c2) Representative MLIF images showing co-labeling of mtG4, Tomm20 and LC3B in PPM within callus at d14 post-fracture in premature progeria mice.

Scale bar, 20 μm. All experiments were technically replicated three times; n = 3 per group.
